# Grey matter atrophy patterns of mobility shared across older adults with and without multiple sclerosis

**DOI:** 10.1007/s00415-025-13373-w

**Published:** 2025-09-26

**Authors:** Mark E. Wagshul, Siddharth Nayak, Frederick W. Foley, Robert W. Motl, Manuel E. Hernandez, Meltem Izzetoglu, Roee Holtzer

**Affiliations:** 1 Department of Radiology, Gruss Magnetic Resonance Research Center, Albert Einstein College of Medicine, 1300 Morris Park Ave., Bronx, NY 10461 USA; 2 Department of Neuroscience, Albert Einstein College of Medicine, Bronx, NY USA; 3 Department of Neurology, Albert Einstein College of Medicine, Bronx, NY USA; 4 Ferkauf Graduate School of Psychology, Yeshiva University, Bronx, NY USA; 5 Multiple Sclerosis Center, Holy Name Medical Center, Teaneck, NJ USA; 6 Department of Kinesiology and Nutrition, University of Illinois at Chicago, Chicago, IL USA; 7 Department of Biomedical and Translational Sciences, Carle Illinois College of Medicine, University of Illinois Urbana-Champaign, Urbana, IL USA; 8 Department of Kinesiology and Community Health, College of Applied Health Sciences, University of Illinois Urbana-Champaign, Urbana, IL USA; 9 College of Liberal Arts & Sciences, Neuroscience Program, University of Illinois Urbana-Champaign, Urbana, IL USA; 10 Beckman Institute, University of Illinois Urbana-Champaign, Urbana, IL USA; 11 Department of Electrical and Computer Engineering, Villanova University, Villanova, PA USA

**Keywords:** Older adults with multiple sclerosis, Structural covariance analysis, Grey matter atrophy, Gait function

## Abstract

Mobility impairment is common in multiple sclerosis, especially in older adults with multiple sclerosis (OAMS). Grey matter (GM) changes are well documented in MS, and GM atrophy is common in older adults. The relationship between GM changes and mobility disability in OAMS is unknown. We sought to identify GM patterns associated with gait speed in OAMS and healthy older controls, using structural covariance network analysis. OAMS (*n* = 102; 64.8 ± 4.4 years) and healthy controls (*n* = 106; 68.2 ± 7.3 years) underwent brain MRI and gait assessments; structural covariance networks were constructed to elucidate brain regions with significant associations between GM volume and 25-foot walk gait speed. We used voxel-wise linear regression analyses to elucidate per-network subregions with significant correlations with gait speed. Voxel-wise moderation analysis tested for group differences in these associations. Across the entire cohort, the following networks demonstrated significant gait speed associations: bilateral hippocampus, bilateral caudate/pallidum/putamen, bilateral thalamus/putamen, right middle temporal gyrus and multiple cerebellar regions. There were no significant group-by-network interaction effects. In summary, structural network analysis reveals unique brain patterns of gait speed in older adults, but these patterns are common amongst healthy older adults and OAMS and highlight the importance of cerebellar and subcortical networks in supporting gait speed.

**Supplementary Information:**

The online version contains supplementary material available at 10.1007/s00415-025-13373-w.

## Introduction

Mobility impairment is a common symptom of multiple sclerosis (MS), a chronic, auto-immune neurodegenerative disease [38], and can be an especially relevant issue in older adults with MS (OAMS) given the overlapping concerns of disease-related and aging-related mobility disability [80]. Many studies have investigated the neurological changes underlying mobility, in healthy aging [40], across multiple clinical populations [54, 63], as well as in younger MS participants [14, 62], but to date there is limited work in OAMS. With the successes of disease-modifying therapies in MS, the aging MS population has grown dramatically, bringing the issues of aging with MS to the forefront.

Early work on neural substrates of MS and MS-related disabilities focused on the white matter pathological changes, but the last 15 years have seen an important shift in this focus toward grey matter and its relationship to MS pathology, clinical outcomes, and management [26, 56]. Whether these grey matter changes drive poor outcomes, are one important component influencing outcome or are merely useful radiological biomarkers incidentally related to functional outcome remains debatable. Nonetheless, large-scale studies demonstrate that grey matter plays an important role in the pathophysiology of MS, at least in young-to-middle-aged adults, and may serve as a valuable marker for predicting clinical outcome and monitoring disease progression [14, 53]. What is less clear is the areas of grey matter most affected, and their association with mobility outcomes. Furthermore, the role of GM in OAMS, where the known effects of grey matter atrophy related to both aging and MS may combine to form a new and perhaps more complex radiological picture, have yet to be adequately explored.

Gait speed is the most studied and validated quantitative measure of gait performance [12, 15, 27, 65, 68, 87]. The decline in gait speed is associated with a multitude of adverse outcomes including but not limited to disability, falls and mortality [66, 73, 91, 99]. Importantly, gait speed serves as primary outcomes in clinical trials and outcome research in MS [21, 29, 49]. A search on www.clinicaltrials.gov revealed over 200 trials in MS with gait velocity as either the primary or secondary outcome in different groups and ages, and timed walking is included in the NIH toolbox for use in clinical trials and epidemiological studies.

With that background, in this work we sought to investigate the brain patterns of grey matter changes associated with gait speed, in OAMS and healthy, older adult controls. We tested the hypothesis that specific grey matter brain patterns are related to walking performance in older adults overall, and that some subset of these patterns may be unique to OAMS compared to age-matched, healthy controls. Specifically, we used structural covariance network (SCN) analysis to explain the grey matter variance in our sample, followed by regression analyses to identify those brain areas associated with gait speed during normal walking, while controlling demographic confounders. Finally, the unique nature of these patterns in OAMS vs. healthy older adults was tested with moderation analyses.

## Methods

### Participants

The sample was drawn from a cohort of older adults, with and without MS, enrolled in a cohort study, “Brain Predictors of Mobility and Falls in Older Adults with Multiple Sclerosis”. Participants, ages 60 and older, were recruited and tested between September 2019 and April 2024; MS participants were recruited from regional treatment centers and patient registry lists and controls from publicly available population lists. Using revised McDonald criteria [92], all patients had physician-confirmed MS diagnoses as determined by medical record review. Both cohorts were screened via a structured telephone interview to obtain verbal consent, screen for dementia, assess medical and psychological history, as well as (self-report) mobility and functional abilities to determine initial study eligibility. All participants were right-handed. Exclusion criteria included diagnosis of major neurological, psychiatric, or medical disease (excluding MS), inability to ambulate independently, MRI contraindications, vision or hearing impairment that would potentially impact testing, and difficulty understanding or speaking English. Presence of peripheral nerve pathology was assessed by questionnaire but was not an exclusion criterion. Cognitive status was determined via established clinical case conference procedures [41]. Participants had two in-person visits, one consisting of a neuropsychological test battery, quantitative gait protocols, and questionnaires, and the second for MRI brain scans and additional questionnaires. On average, the second visit was 5.8 days after the first visit (Note: during the COVID-19 pandemic, there were unavoidable scheduling delays resulting in 6 participants with 1–2 months and 2 participants with 7 months between visits). The study was approved by the IRB of Albert Einstein College of Medicine (IRB Protocol #2019–10049) and all participants provided written informed consent prior to enrollment. The work described in this manuscript was executed in adherence with The Code of Ethics of the World Medical Association (Declaration of Helsinki).

### Structural image acquisition

MRI was performed in a 3 T Philips scanner equipped with a 32-channel head coil (Elition 3.0 T X, Philips Medical Systems, Best, The Netherlands) at the Gruss MR Research Center of Albert Einstein College of Medicine. High resolution 3D T1-weighted (MPRAGE) structural brain images were acquired for GM quantification (see below), and to identify T1-hypointense MS lesions (TE/TR/TI: 4.6/9.7/900 ms, voxel size: 1 mm isotropic, flip angle: 8°). 3D fluid-attenuated inversion recovery (FLAIR) images were acquired for identification of hyper-intense T2 lesions (TE/TR/TI: 365/4800/1650 ms, voxel size: 1 mm isotropic); both FLAIR and T1 images were used to identify and quantify MS lesions using the lesion segmentation toolbox [82]. Spinal cord volume, shown in previous literature to be a significant predictor of physical disability in MS [94], was segmented from FLAIR images, similar to prior work [4] using a 35-mm segment of the spinal cord, starting 27-mm below the prepontine cistern.

The Computational Anatomy Toolbox, version 12 (CAT12) was used to perform voxel-based morphometry and construction of the structural networks [2, 32]. The algorithm uses geodesic shooting [5] to register the individual tissue segments to a standard template (GM tissue probability map, 1.5 mm isotropic), and the registration parameters were used to calculate local grey matter volume on a pixel-by-pixel basis based on modulation of the local gray matter probability with the Jacobian of the subject-to-template registration warp field[58]; note that all calculations were performed in this study-specific space, and when needed, linear registration to MNI space was used for anatomical region identification. GM volume maps were smoothed with a 3-sigma gaussian kernel to accommodate differences in grey matter anatomy and registration. Following registration, grey matter networks were decomposed into twenty (20) components, which was adequate to represent the major brain networks; increasing the number of networks beyond 20 runs the risk of unnecessary splitting of individual networks into multiple components. 

### Regression analysis

By combining brain regions into a single network, the above analysis attempts to mitigate type I errors associated with statistical analysis across a large set of measurements, such as with whole-brain voxel-wise analysis. On the other hand, the structural network reduction described above deconstructs the brain into not only a specific collection of voxels (*i.e.,* those making up the network), but also a very specific relationship between those voxels (*i.e.,* within-network loadings). However, in the investigation of the relationship between these networks and some outcome measure, this specific network construct has the potential for type II errors (*i.e.,* each network as a whole may adequately characterize the entire subject cohort but does not necessarily adequately characterize the relationship between the network and the outcome measure). For example, there may be a strong relationship between the outcome measure with only parts of the network and reducing the per-subject representation of the network to a single per-subject score, as is commonly practiced, can dramatically attenuate this relationship.

Thus, as a compromise between these two approaches, we chose a middle ground approach. First, SCN was used to identify collections of voxels with common variance across the entire cohort. Voxels within this network (at the level of *t* ≥ 2) were then used as a mask for voxel-wise linear regression analyses, to identify which portions of each network demonstrate a relationship between GM volume and gait speed (see, e.g. [35]). Linear regressions were carried out with the FSL “randomise” package [96], with threshold-free cluster enhancement and 5000 permutations to calculate family-wise error corrected statistical significance. All models were run with age, sex, years of education, global health scale (see below) and total intracranial volume as covariates. All variables were mean centered and standardized prior to being entered into the randomise design matrix.

### Moderation analysis—group differences

To investigate potential differences between MS and control (CTL) participants in the associations between GM volume and gait speed, we conducted a moderation analysis with the addition of a group-by-gait speed interaction term in the linear regressions. Two interaction analyses were used to test this hypothesis both 1) across each entire network and 2) within areas of significant linear regression as identified above from the entire cohort. In the first instance, voxel-wise regression analyses were used across each of the 20 structural networks, similar to those described above, with the addition of an interaction term of group status (CTL/MS = 0/1) * timed 25-foot walk (T25FW) gait speed. In the second instance, for each network with significant associations across the entire cohort identified in the analysis above, we calculated mean grey matter volume within the mask of significant voxels and ran linear regressions for these mean grey matter volumes as follows:

$$Mean GM volume={\beta }_{0}+{\beta }_{1}*T25FW+{\beta }_{2}*Group+{\beta }_{3}*Group*T25FW+{\beta }_{4-8} *Covariates$$

### Physical function predictor measure—gait speed

Gait speed (ft/s) was measured through the timed 25-foot walk, which was administered over two trials according to standard procedures [59]; total time was taken as the average of the two trials and converted to velocity in feet/second. Participants were instructed to walk at their normal, comfortable pace. Gait speed has proven as a robust measure of physical function in both healthy aging [95] and with disease [57], and has served as a proxy for disability in MS in numerous studies [6, 36, 39, 59, 60].

### Alternate outcome measures

To appreciate the significance of our results with respect to the relationship of grey matter patterns to walking performance, we explored the association of gait speed with other common measures which may play roles in MS, in older adult populations and in gait, including: Total grey matter volume (GMV) [44], total white matter volume (WMV) [53], normal appearing white matter volume (NAWM) [56], white matter lesion load (LL) [85] and spinal cord volume (SCV) [93].

### Covariates

Covariates included: age, sex, years of education and Global Health Status (GHS). The GHS scale included presence/absence of the following conditions (max score 10): diabetes, chronic heart failure, arthritis, hypertension, depression, stroke, Parkinson’s disease, chronic obstructive lung disease, angina, and myocardial infarction [41].

### Other measures used to characterize participants

*RBANS*: This is a well-validated battery of 12 subtests spanning five cognitive domains: immediate memory, visuospatial/constructional abilities, language, attention, and delayed memory [75]. The RBANS total index score has been used as a proxy for the general level of cognitive function.

*PDDS*: MS-related disability was measured in the patient group only with the 8-point, self-reported PDDS scale, which has been shown to strongly correlate with the gold standard Expanded Disability Status Scale (EDDS) [51] and recently validated for OAMS [52]. PDDS scores range from 0 (no disability) to 8 (bedridden).

### Statistical analysis

Continuous demographic, clinical and imaging measures were compared between the experimental groups using independent t-statistics; in cases of non-normal data distributions, Wilcoxon rank sum tests were used. To address the main hypothesis of the manuscript that specific brain networks are associated with the functional measure of gait speed, models explored the linear relationship between pattern scores and gait speed, as follows:

$$GM volume={\beta }_{0}+{\beta }_{1}*T25FW+{\beta }_{2-6} *Covariates$$

Statistical analyses were carried out with FSL’s randomise (FSL version R6.0.5), with threshold-free cluster enhancement and 5000 permutations, and all statistical tests were considered significant at *p* < 0.05. Only clusters with a minimum extent of 10 voxels were maintained.

### Sensitivity analyses

Because of the complex interdependence between grey matter structural network integrity and other potential measures of brain integrity, sensitivity analyses tested whether grey matter network measures were related to gait speed *independent* of these other measures. Separate models were run for networks that were significantly associated with gait speed adjusting for each alternate measure of brain integrity, restricted to those measures demonstrating highly significant relationships to T25FW (NAWM, LL and SCV).

We further explored whether MS-related disability levels (mild: PDDS = 0–2 vs moderate: PDDS = 3–5, [48]) influenced associations between networks and T25FW.

## Results

### Participants

From an initial pool of 222 participants (110 OAMS) who completed the imaging portion of the study, eight MS participants were excluded (four with a dementia diagnosis, one with missing gait data and three with registration issues) and six control participants were excluded (four with a dementia diagnosis, and two with registration issues). Thus, the final cohort consisted of 208 participants, 102 OAMS and 106 healthy controls. The MS participants were on average younger (MS: 64.8 ± 4.3 vs CTL: 68.2 ± 7.2 years, *p* < 0.0001) and reported fewer years of education (MS: 15.2 ± 2.4 vs CTL: 16.4 ± 2.5 years, *p* = 0.0008). Global health scores indicated an overall small number of comorbidities, with no group difference (*p* = 0.29) and RBANS scores indicated an overall average cognitive status, with MS participants scoring slightly below controls (MS: 89.5 ± 12.0 vs CTL: 93.3 ± 12.9, *p* = 0.025). Median PDDS (MS participants only) was 2, with a range of 0–5, indicating that our MS participants reported mild to moderate disability. MS participants had slightly lower total grey matter volume (MS: 400.7 ± 41.8 vs CTL: 416.5 ± 43.4 cm^3^, *p* = 0.008, *d* = 0.37), and lower total NAWM volume (MS: 444.2 ± 52.9 vs CTL: 471.9 ± 98.1 cm^3^, *p* = 0.008, *d* = 0.37). Finally, with respect to the gait data, MS participants on average had slower gait speed during normal walking (MS: 4.04 ± 1.1 vs CTL: 4.69 ± 0.95 feet/sec, *p* < 0.0001, *d* = 0.60). Demographic, clinical, and volumetric results and comparisons can be found in Table 1.

**Table 1:** Demographic, clinical, brain imaging and walking measures

	OAMS (*n* = 102)	HC (*n* = 106)	*p* (OAMS vs HC)
Demographic			
Age (years)	64.8 ± 4.3 (60–76)	68.2 ± 7.3 (60–91)	**< 0.0001**
Gender (F/M, n)	73/29	71/35	0.57
Education (years)	15.2 ± 2.4 (11–21)	16.4 ± 2.4 (10–21)	**0.0008**
GHS	1 (0–2, range: 0–5)	1 (1–2, range: 0–4)	0.90
RBANS	89.5 ± 12.0 (62–120)	93.3 ± 12.9 (65–124)	0.025
PDDS	2 (1–4, range: 0–5)		
MS subtype (n)			
Relapsing–remitting	71		
Secondary-progressive	19		
Primary-progressive	7		
Not known	5		
Brain measures (cc)			
T2 lesion volume	12.06 ± 11.06	4.48 ± 6.45	** < 0.0001**
Intracranial volume	1327.7 ± 141.5	1355.3 ± 136.7	0.15
Total GM volume	400.7 ± 41.8	416.5 ± 43.4	**0.008**
Total WM volume	456.0 ± 52.3	467.9 ± 51.1	0.097
Total NAWM	444.2 ± 52.9	463.6 ± 51.4	**0.008**
Spinal cord volume	2.70 ± 0.29	2.94 ± 0.30	**< 0.0001**
Gait measure			
Walking speed (ft/s)	4.04 ± 1.11	4.69 ± 0.95	**< 0.0001**

Values represent mean ± SD (range), or median (IQR) for GHS and PDDS. *p*-values < 0.05 shown in bold

*OAMS* older adults with multiple sclerosis, *HC* healthy older controls, *GHS* global health score, *GM* grey matter, *NAWM* normal appearing white matter, *PDDS* patient determined disease steps, *RBANS* repeatable battery for the assessment of neuropsychological status, *WM* white matter

### VBM

**Fig. 1:**
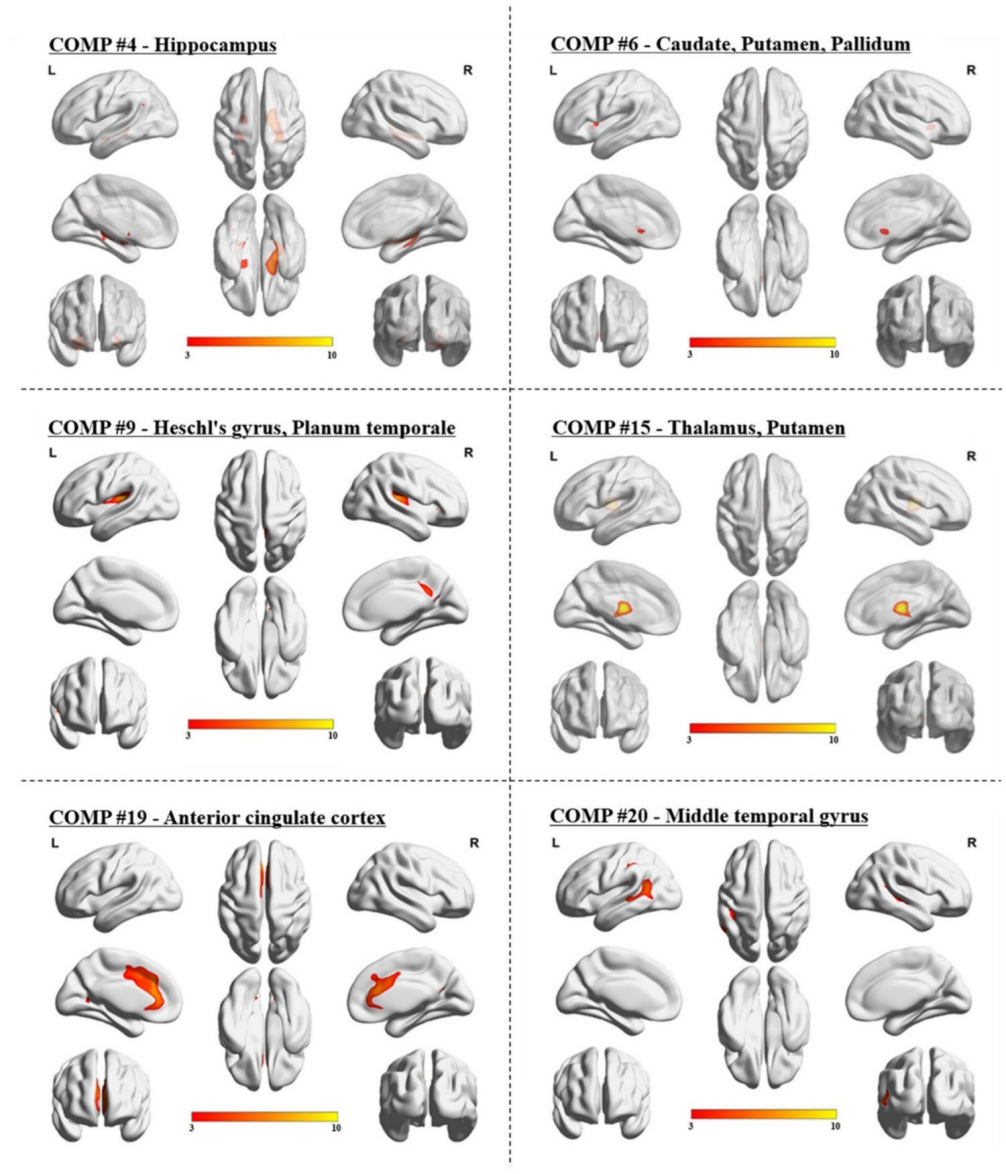
Structure covariance networks demonstrating significant differences between MS participants and healthy controls, adjusted for demographic variables and total intracranial volume. Note: group differences were tested on per-subject *network expression* values and therefore cannot elucidate group differences within the network. Color codes therefore do not represent group difference values but rather represent the across network expression values of the networks themselves dictated by the CAT12 analyses

Voxel-based morphometry isolated 20 unique networks, covering all brain regions (see Supplementary Fig. 1) and was consistent with previous structural covariance findings [17, 74]. Of note, but also consistent with previous SCN literature, is the fine parcellation of the cerebellum, which encompassed 5 of the 20 structural networks. Networks partially overlapped with prior studies of structural covariance and functional connectivity network studies, covering parts of the medial visual, auditory, motor, posterior default mode, dorsal attention, semantic and basal ganglia networks [76, 81, 97, 98]. CAT12 identified 6 networks with significant differences between MS participants and controls (see Fig. 1: hippocampus, caudate/pallidum/putamen, thalamus/putamen, parietal operculum/Heschl’s gyrus/insula, anterior cingulate gyrus, middle temporal gyrus (MTG)—all networks were bilateral); in all significant networks, grey matter volumes were *lower* in MS compared to controls (*p* < 0.05).

### Regression model results

**Fig. 2:**
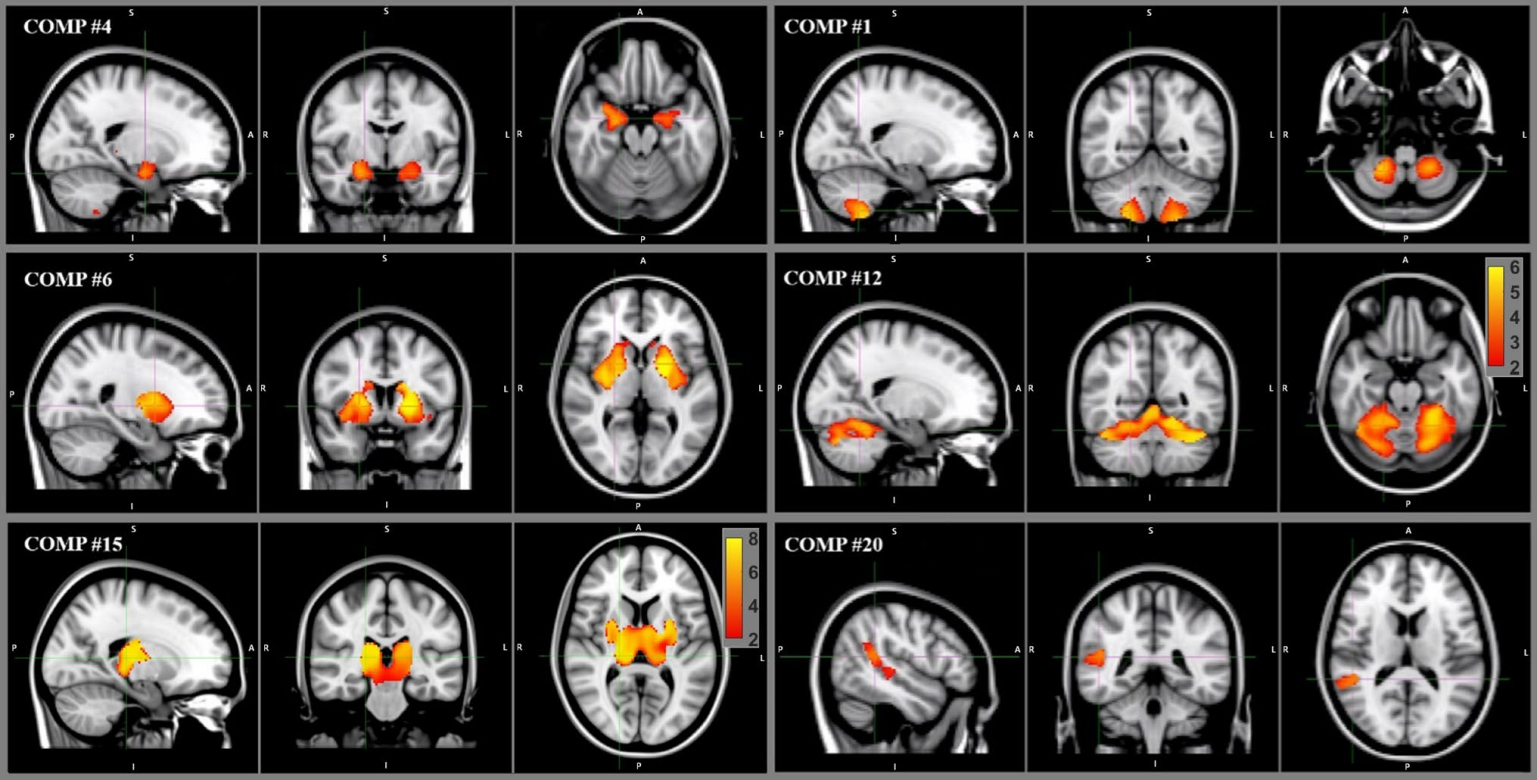
Network-wise regression maps, showing areas with significant relationship between grey matter volume and gait speed (values shown are -log(p); note the alternate scale for the thalamus component, lower left panel). Note: Only two representative cerebellar networks are shown.

Slower T25FW gait speed was significantly associated with smaller grey matter volume in the following networks (see Fig. 2): cerebellum (multiple networks encompassing areas V, VI VIIIb, and Crus I), bilateral amygdala/hippocampus, bilateral posterior caudate/putamen/insula, bilateral thalamus/putamen, and right MTG/supramarginal/angular gyrus. There were no areas in which slower gait speed was associated with increased grey matter volume. Full model results, including peak cluster identifications and locations and can be found in Table 2.

**Table 2:** Regions demonstrating significant within-network associations between grey matter volume and the dependent variable, the Timed 25-foot walk normal walking speed

Cluster index	# Voxels	Max_X (vox)	Max_Y (vox)		Max_Z (vox)	Anatomical regions
Network #1	467	56	37	5		L-Cerebellum—Lobule VIII
	420	34	37	6		R-Cerebellum—Lobule VIII
Network #2	148	65	41	21		L-Cerebellum—Lobule VI
Network #4	296	31	58	30		L-Hippocampus, Amygdala
	99	57	59	27		R-Amygdala
Network #5	18	54	35	29		L-Cerebellum—Lobule VI
Network #6	1482	55	62	39		L-Caudate, Pallidum. Putamen
	1394	35	61	39		R-Pallidum. Putamen
	74	36	58	48		R-Caudate
Network #11	124	33	37	8		L-Cerebellum—Lobule VIII
	89	56	37	7		R-Cerebellum—Lobule VIII
Network #12	3934	64	39	21		L-Cerebellum—Lobule I-IV, V, VI, Crus I
						R-Cerebellum—Lobule V, VI, Crus I
Network #13	205	34	38	7		R-Cerebellum—Lobule VIII
Network #14	501	64	39	21		L-Cerebellum—Crus I
	89	53	34	29		L-Cerebellum—Lobule VI
Network #15	4563	36	57	38		L-Thalamus, Putamen
						R-Thalamus, Putamen
Network #16	535	64	39	21		L-Cerebellum—Crus I, Lobule VI
	80	56	38	7		R-Cerebellum—Lobule VIII
Network #17	12	33	60	27		R-Amygdala
Network #18	939	22	36	23		R-Cerebellum—Crus I
	921	64	39	21		L-Cerebellum—Crus I
Network #20	143	21	42	44		R-Angular gyrus
						R-Middle temporal gyrus
						R-Supramarginal gyrus

The magnitude of the regression relationships can help us appreciate the significance of the findings and the potential role of the networks discovered in maintaining or Supporting gait function in older adults with and without MS. The significant associations demonstrated a relatively small range in slopes, from 0.266 to 0.371 ft/sec change in gait speed per 1 standard deviation change in GM volume. These numbers are consistent with the low-to-mid range of the minimal clinically important difference previously reported in older adults and in MS and related disorders [9, 50], indicating that the differences seen in GM volume may have a clinically meaningful impact on outcome measures such as gait function in older adults with or without MS.

### Moderation analysis

**Fig. 3:**
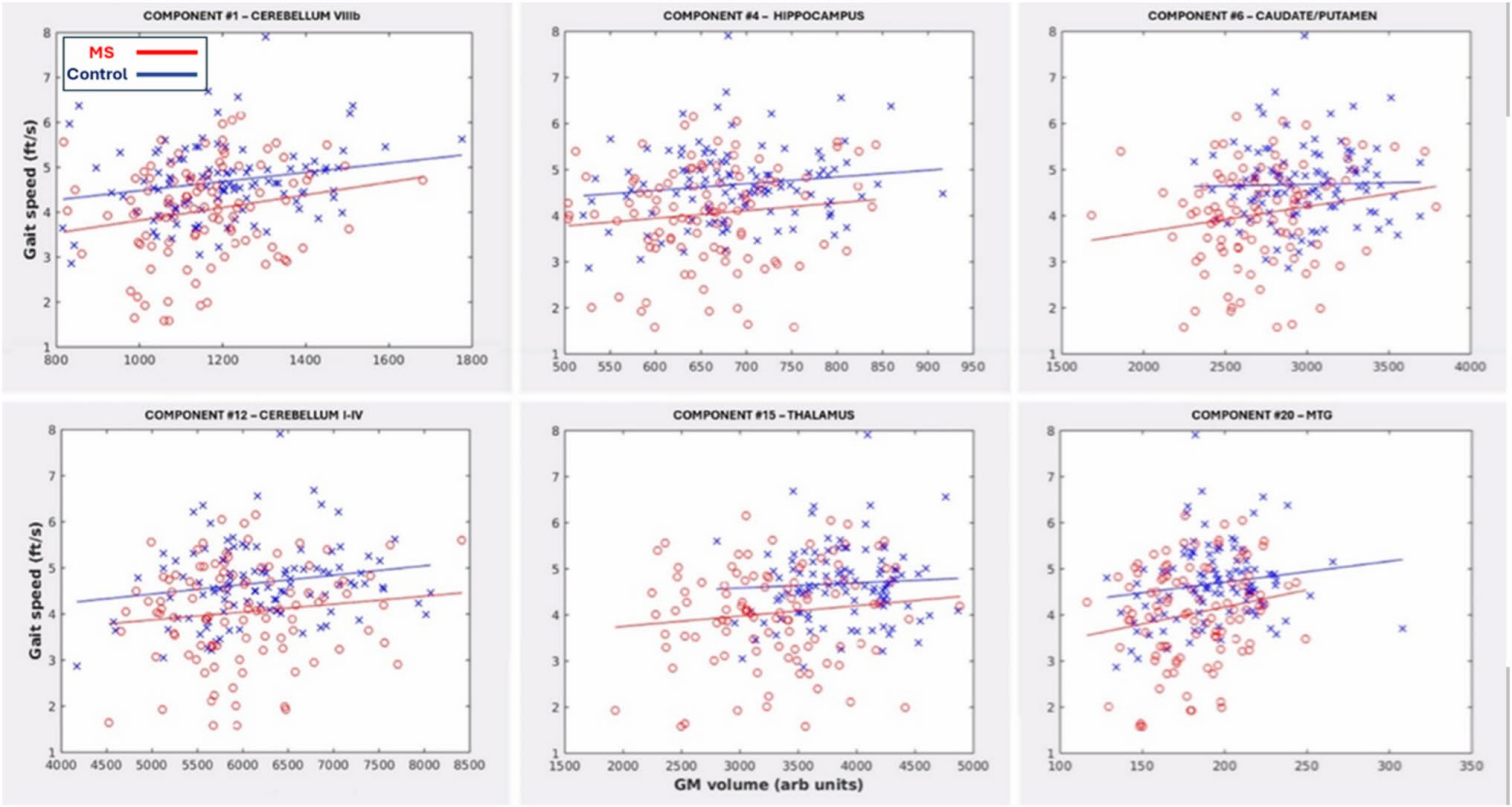
Scatter plots for each of the significant networks, showing mean GM volume within significant portions of the networks vs. T25FW gait speed, stratified by study group

None of the moderation models were significant. Across networks with significant associations across the entire cohort, GM volume-gait speed associations were similar between the two groups, with no significant interaction terms (see Fig. 3).

### Alternate predictor and sensitivity analyses

As independent predictors of gait speed, total WM, NAWM and SCV were positively associated with gait speed (total WM: *p* = 0.043; NAWM: *p* = 0.0009; SCV: *p* = 0.0002), and whole brain WM lesion load was negatively associated with gait speed (LL: *p* = 0.0004), while total GM volume was not a significant predictor of gait speed (total GM: *p* = 0.089). The significant predictors corresponded to slopes of 0.386 (WM), 0.568 (NAWM), 0.294 (SCV) and −0.250 (LL) ft/sec change in gait speed per 1 standard deviation unit increase in predictor score. When entered simultaneously into models together with mean component scores, NAWM, LL and SCV were significant predictors in most models. Importantly, component scores remained as significant *independent* predictors of gait speed in all cases (see Supplemental Tables 1–3).

MS-related disability: In participants with mild disability (PDDS = 0–2, *n* = 55), four GM networks showed significant associations with gait speed and five showed associations at the trend level, including cerebellum, striatum and hippocampus. In contrast, those with moderate disability (PDDS = 3–5, *n* = 47) demonstrated only one network with trend-level association with gait speed (*p* = 0.09, right STG) while all other networks showed no associations (*p* > 0.27). These analyses are summarized in Supplemental Table 4.

## Discussion

The current study investigated latent structural grey matter networks in order to identify patterns of brain atrophy implicated in walking ability, in older adults with and without multiple sclerosis. Three main conclusions were reached: (1) Structural covariance networks extracted across the entire cohort matched patterns demonstrated both in older healthy controls and in MS participants, with almost exclusively bilateral networks, and representing cerebellum, and subcortical grey matter, and cortical networks across frontal, parietal, temporal and occipital lobes, (2) network differences between healthy older adults and MS align with prior findings in younger participants and in particular clearly accentuating subcortical network atrophy in MS and (3) across the entire cohort, the relationship between grey matter atrophy and slower gait speed highlighted the importance of cerebellum, subcortical structures and the right middle temporal gyrus in support of gait speed in older adults with and without MS. Importantly, however, the effect of group on these relationships was not significant, indicating that associations of grey matter atrophy within these networks with gait performance were shared among OAMS and controls. These findings build on and extend prior publications reporting subcortical grey matter atrophy in MS [11, 19, 26, 46], as well as the relationship between physical function and subcortical atrophy in both younger [6, 67, 83] and older MS participants [8, 62].

### Prior findings—structural differences in MS

Group comparisons of the structural covariance networks identified multiple subcortical regions with reduced grey matter in MS participants compared to controls, with the most significant differences in the thalamus. This has been shown in earlier publications, although most studies were in middle-aged adults [71, 77, 83]. A recent longitudinal study charted the changes in subcortical structures in MS patients from 20 to 60 years old compared to controls, demonstrating the most dramatic volume reductions in thalamus, followed by putamen and pallidum, also in line with our findings [20]. In cortex, prior studies have demonstrated atrophy within the anterior cingulate cortex [1, 16, 17], MTG [17, 70, 86] and insula [16, 84]. Thus, our findings have good support from prior literature, as well as extending these findings into the OAMS population.

### Prior findings—relationship of GM volume to gait function

Networks associated with normal walking gait speed highlighted subcortical regions, cerebellum and the right MTG/supramarginal gyrus. Gait studies in healthy older adults have found significant associations with gait speed in thalamus [3, 8, 43, 62, 72, 88], caudate [25, 61], pallidum [61], hippocampus [28, 79] and MTG [24, 72]. Cerebellar regions are often not included in these studies, but a few studies have also reported associations with cerebellar volumes [13, 31]. In OAMS, mostly subcortical areas have demonstrated significant associations with gait speed [8, 61]. One study demonstrated limited areas of association of cerebellar grey matter atrophy with gait speed [34]. However, it is important to point out that two of these studies did not include a control group [34, 61], while the third demonstrated associations of gait speed with subcortical GM atrophy across both the MS and control groups [8]. Thus, earlier works have not addressed the question of differences in network associations between OAMS and healthy older adults. While we cannot rule out the possibility that our study lacked power to demonstrate these differences, our moderation analyses as well as the scatter plots shown in Fig. 3 support common relationships in all these networks across both healthy older adults and MS participants.

### Interpretation of findings

The five broad regions identified in this study encompass thalamus, caudate/pallidum/putamen, hippocampus, cerebellum and middle temporal gyrus. Interestingly, findings on the relative importance of basal ganglia structures in comparison to the thalamus regions are mixed, with findings of involvement of caudate and/or putamen to the exclusion of thalamus [61], thalamus to the exclusion of basal ganglia structures [83], or combinations of the two [62]. Our analyses did not allow for multiple regressions across brain networks; extremely high multicollinearity between these networks (*r* = 0.75) would preclude inclusion of both measures in a single model. Nonetheless, both thalamus and basal ganglia structures generally demonstrate strong univariate relationships with gait performance across many studies and clinical populations [8, 10, 18, 88, 90]. The hippocampus is vulnerable to atrophy with aging [30] and in multiple sclerosis [6, 7] and has been associated with cognitive impairments. The right hippocampus, associated with spatial memory and navigation [47, 64], demonstrated a threefold larger area of association between GM volume and gait speed, compared to the left hippocampus. The cerebellum plays multiple roles in gait function, in particular with respect to balance, and has been shown previously to be related to gait speed [13, 78] and other gait functions [45], and in the context of multiple sclerosis and gait function [34]. While our data did not highlight associations within frontal lobe, possibly because of the relatively simple walking task utilized, there is extensive literature on the involvement of cerebello-thalamic-frontal pathways [33, 42, 69], and would seem to be at least partly involved through the associations demonstrated in cerebellar and subcortical regions. Finally, the middle temporal gyrus plays a role in multimodal sensory integration and spatial awareness. Doi and colleagues, using structural network analysis in patients with mild cognitive impairment, identified multiple grey matter networks associated with dual-task gait performance, with the largest region of association in middle temporal gyrus [24]. Another study, also in mild cognitive impairment, similarly found areas of association between grey matter atrophy and the 6 m walk gait speed in right middle temporal gyrus and hippocampus [55]. Again, most notably, our work highlights the fact that the involvement of these regions in gait performance would appear to be shared across older adults with and without MS.

The spinal cord has a key role in mobility-disability in MS, although it has not been investigated in OAMS. Interestingly, spinal cord atrophy displayed one of the strongest differences between study groups, and we therefore sought to determine whether or not SCV attenuated relationships of the GM network to walking speed. Our sensitivity analyses did show a strong relationship between SCV and gait speed, data which are in line with prior findings in younger adults with MS [94]. While inclusion of component GM volume and SCV in the same regression models did weaken the GM component-gait relationship, both GM and SCV remained as *independent* predictors of motoric outcome, with similar magnitude. Notably, standardized betas indicate that GM volume within mostly subcortical and cerebellar networks play a role comparable to the role of spinal cord atrophy in predicting gait function in older adults both with and without MS.

On the issue of the clinical significance of our findings, we can ask the question in this way: do reasonable network changes (*e.g.*, 1 standard deviation change in mean network GM volume) translate into clinically meaningful changes in physical function? From amongst the whole brain measures, total WM and NAWM volumes provided the best prediction capabilities, and superior to any of the GM network predictions. Both of these measures would qualify as clinically significant changes, according to a recent study investigating multiple gait-related outcomes in MS [50]. Nonetheless, the clear advantage of localized grey matter network measures over a whole brain approach is the potential for using brain locations to arrive at a mechanistic understanding of the relevance of imaging changes to function outcome.

Finally, stratified analysis showed that GM component-gait speed associations were observed exclusively in OAMS with mild disability. It may be that while GM network integrity controls gait performance in healthy and mildly disabled individuals, this function breaks down with more advanced disease processes. However, because these analyses required cutting our sample size by a factor of four, we cannot rule out the possibility that the lack of significance stems from a lack of adequate power to demonstrate significant associations in both sub-groups.

### Strengths and limitations

We present compelling evidence for shared cerebellar and subcortical networks that support gait speed in OAMS and control participants. The study was carried out in a large cohort with over 100 participants per group, lending further support to the robustness of the findings. It is possible that the relatively simple nature of the walking task led to these common findings between groups; analyses of structural networks supporting more complex walking tasks, such as dual-task walking, will be the focus of future work. The OAMS were overall slightly but significantly younger and had fewer years of education compared to the controls, which may have affected the comparisons between the groups. However, all analyses were corrected for age and education, and the main effects were mostly unchanged. Notably, if anything, the younger age of the MS participants may have skewed our data in the direction of a smaller effect on grey matter loss. Our MS participants were not severely disabled, as shown by the relatively low PDDS scores. This was a product of the inclusion criteria of the parent study, which required participant to be ambulatory. This may limit the generalizability of our findings and future studies with more impaired participants should be conducted. Finally, while analyses adjusted for SCV, we did not adjust for spinal cord lesions.

## Conclusion

We present evidence for structural brain networks of walking, patterns that are shared across older adults with and without MS. These findings might be used in future studies to investigate the structural brain networks that support mobility function in other clinical populations, or as a surrogate, objective marker of disease progression or improvement through therapeutic interventions.

## Supplementary Information

Below is the link to the electronic supplementary material.

Supplementary file1 (DOCX 43 KB)

## Data Availability

Data will be provided to qualified investigators upon reasonable written request to the corresponding author.
