# Supplementary material for: Grey matter atrophy patterns of mobility shared across older adults with and without multiple sclerosis
Source: J Neurol. 2025 Sep 26;272(10):656. doi: 10.1007/s00415-025-13373-w (PMC12474737; doi:10.1007/s00415-025-13373-w)
Supplement: Supplementary file 1 — Supplementary file1 (DOCX 43 KB) [file 415_2025_13373_MOESM1_ESM.docx]

**Table S1: Effect of spinal cord volume on models:** Statistical results from GM component models, exploring the effects of spinal cord volume on regression results; outcome variable for all models was the Timed 25-foot walk normal walking speed. The first line for each component model indicates results for linear regression against GM volume scores alone, while the second and third line indicate results for models including both GM volume and SCV as predictors.

| **NETWORK #** | **Parameter** | **B** | | | **SE** | **Standardized B** | | | **p-value** |
| --- | --- | --- | --- | --- | --- | --- | --- | --- | --- |
| Network #1 | GM volume only | | 1.881 | 0.481 | | | 0.308 | **0.0001** | |
|  | GM volume | | 1.580 | 0.480 | | | 0.259 | **0.0012** | |
|  | SCV | | 0.761 | 0.240 | | | 0.244 | **0.0018** | |
| Network #2 | GM volume only | | 8.348 | 2.082 | | | 0.337 | **0.0001** | |
|  | GM volume | | 6.891 | 2.096 | | | 0.278 | **0.0012** | |
|  | SCV | | 0.736 | 0.242 | | | 0.236 | **0.0026** | |
| Network #4 | GM volume only | | 4.689 | 1.240 | | | 0.367 | **0.0002** | |
|  | GM volume | | 3.488 | 1.298 | | | 0.273 | **0.0078** | |
|  | SCV | | 0.687 | 0.252 | | | 0.220 | **0.0071** | |
| Network #5 | GM volume only | | 72.180 | 18.277 | | | 0.346 | **0.0001** | |
|  | GM volume | | 56.102 | 18.880 | | | 0.269 | **0.0033** | |
|  | SCV | | 0.692 | 0.248 | | | 0.222 | **0.0058** | |
| Network #6 | GM volume only | | 0.942 | 0.236 | | | 0.331 | **0.0001** | |
|  | GM volume | | 0.735 | 0.244 | | | 0.258 | **0.0029** | |
|  | SCV | | 0.687 | 0.248 | | | 0.220 | **0.0063** | |
| Network #11 | GM volume only | | 5.232 | 1.291 | | | 0.318 | **0.0001** | |
|  | GM volume | | 4.408 | 1.291 | | | 0.268 | **0.0008** | |
|  | SCV | | 0.751 | 0.240 | | | 0.240 | **0.0020** | |
| Network #12 | GM volume only | | 0.426 | 0.119 | | | 0.324 | **0.0004** | |
|  | GM volume | | 0.323 | 0.122 | | | 0.246 | **0.0086** | |
|  | SCV | | 0.728 | 0.248 | | | 0.233 | **0.0037** | |
| Network #13 | GM volume only | | 5.850 | 1.475 | | | 0.316 | **0.0001** | |
|  | GM volume | | 4.930 | 1.472 | | | 0.266 | **0.0010** | |
|  | SCV | | 0.759 | 0.240 | | | 0.243 | **0.0018** | |
| Network #14 | GM volume only | | 2.811 | 0.667 | | | 0.359 | **0.0000** | |
|  | GM volume | | 2.265 | 0.684 | | | 0.289 | **0.0011** | |
|  | SCV | | 0.686 | 0.246 | | | 0.220 | **0.0057** | |
| Network #15 | GM volume only | | 0.456 | 0.123 | | | 0.267 | **0.0003** | |
|  | GM volume | | 0.347 | 0.127 | | | 0.203 | **0.0068** | |
|  | SCV | | 0.715 | 0.249 | | | 0.229 | **0.0044** | |
| Network #16 | GM volume only | | 2.848 | 0.647 | | | 0.371 | **0.0000** | |
|  | GM volume | | 2.362 | 0.658 | | | 0.307 | **0.0004** | |
|  | SCV | | 0.692 | 0.242 | | | 0.222 | **0.0047** | |
| Network #17 | GM volume only | | 73.177 | 20.056 | | | 0.336 | **0.0003** | |
|  | GM volume | | 53.458 | 20.986 | | | 0.246 | **0.0116** | |
|  | SCV | | 0.698 | 0.253 | | | 0.224 | **0.0063** | |
| Network #18 | GM volume only | | 0.880 | 0.238 | | | 0.314 | **0.0003** | |
|  | GM volume | | 0.680 | 0.243 | | | 0.243 | **0.0056** | |
|  | SCV | | 0.723 | 0.247 | | | 0.232 | **0.0038** | |
| Network #20 | GM volume only | | 10.172 | 2.682 | | | 0.295 | **0.0002** | |
|  | GM volume | | 8.477 | 2.676 | | | 0.245 | **0.0018** | |
|  | SCV | | 0.766 | 0.241 | | | 0.245 | **0.0017** | |

SCV: Spinal cord volume.

**Table S2:** **Effect of NAWM volume on models:** Statistical results from GM component models, exploring the effects of normal appearing white matter volume on regression results; outcome variable for all models was the Timed 25-foot walk normal walking speed. The first line for each component model indicates results for linear regression against GM volume scores alone, while the second and third line indicate results for models including both GM volume and NAWM as predictors.

| **NETWORK #** | **Parameter** | **B** | | | **SE** | **Standardized**  **B** | | | **p-value** |
| --- | --- | --- | --- | --- | --- | --- | --- | --- | --- |
| Network #1 | GM volume only | | 1.881 | 0.481 | | | 0.308 | **0.0001** | |
|  | GM volume | | 1.707 | 0.475 | | | 0.279 | **0.0004** | |
|  | NAWM | | 0.009 | 0.003 | | | 0.496 | **0.0029** | |
| Network #2 | GM volume only | | 8.348 | 2.082 | | | 0.337 | **0.0001** | |
|  | GM volume | | 6.608 | 2.230 | | | 0.267 | **0.0034** | |
|  | NAWM | | 0.007 | 0.003 | | | 0.369 | **0.0396** | |
| Network #4 | GM volume only | | 4.689 | 1.240 | | | 0.367 | **0.0002** | |
|  | GM volume | | 3.523 | 1.367 | | | 0.275 | **0.0106** | |
|  | NAWM | | 0.007 | 0.003 | | | 0.362 | 0.0507 | |
| Network #5 | GM volume only | | 72.180 | 18.277 | | | 0.346 | **0.0001** | |
|  | GM volume | | 58.533 | 19.004 | | | 0.281 | **0.0024** | |
|  | NAWM | | 0.008 | 0.003 | | | 0.403 | **0.0208** | |
| Network #6 | GM volume only | | 0.942 | 0.236 | | | 0.331 | **0.0001** | |
|  | GM volume | | 0.727 | 0.263 | | | 0.255 | **0.0063** | |
|  | NAWM | | 0.006 | 0.004 | | | 0.334 | 0.0735 | |
| Network #11 | GM volume only | | 5.232 | 1.291 | | | 0.318 | **0.0001** | |
|  | GM volume | | 4.694 | 1.281 | | | 0.286 | **0.0003** | |
|  | NAWM | | 0.009 | 0.003 | | | 0.481 | **0.0040** | |
| Network #12 | GM volume only | | 0.426 | 0.119 | | | 0.324 | **0.0004** | |
|  | GM volume | | 0.322 | 0.126 | | | 0.245 | **0.0117** | |
|  | NAWM | | 0.008 | 0.003 | | | 0.401 | **0.0256** | |
| Network #13 | GM volume only | | 5.850 | 1.475 | | | 0.316 | **0.0001** | |
|  | GM volume | | 5.324 | 1.457 | | | 0.287 | **0.0003** | |
|  | NAWM | | 0.009 | 0.003 | | | 0.496 | **0.0029** | |
| Network #14 | GM volume only | | 2.811 | 0.667 | | | 0.359 | **0.0000** | |
|  | GM volume | | 2.254 | 0.726 | | | 0.288 | **0.0022** | |
|  | NAWM | | 0.006 | 0.003 | | | 0.340 | 0.0610 | |
| Network #15 | GM volume only | | 0.456 | 0.123 | | | 0.267 | **0.0003** | |
|  | GM volume | | 0.323 | 0.147 | | | 0.189 | **0.0289** | |
|  | NAWM | | 0.006 | 0.004 | | | 0.327 | 0.1025 | |
| Network #16 | GM volume only | | 2.848 | 0.647 | | | 0.371 | **0.0000** | |
|  | GM volume | | 2.349 | 0.690 | | | 0.306 | **0.0008** | |
|  | NAWM | | 0.007 | 0.003 | | | 0.349 | **0.0485** | |
| Network #17 | GM volume only | | 73.177 | 20.056 | | | 0.336 | **0.0003** | |
|  | GM volume | | 55.217 | 21.515 | | | 0.254 | **0.0110** | |
|  | NAWM | | 0.007 | 0.003 | | | 0.391 | **0.0306** | |
| Network #18 | GM volume only | | 0.880 | 0.238 | | | 0.314 | **0.0003** | |
|  | GM volume | | 0.677 | 0.252 | | | 0.242 | **0.0079** | |
|  | NAWM | | 0.007 | 0.003 | | | 0.395 | **0.0272** | |
| Network #20 | GM volume only | | 10.172 | 2.682 | | | 0.295 | **0.0002** | |
|  | GM volume | | 7.979 | 2.828 | | | 0.231 | **0.0053** | |
|  | NAWM | | 0.007 | 0.003 | | | 0.397 | **0.0253** | |

NAWM: Normal appearing white matter.

**Table S3:** **Effect of WM lesion load on models:** Statistical results from GM component models, exploring the effects of total white matter lesion load volume on regression results; outcome variable for all models was the Timed 25-foot walk normal walking speed. The first line for each component model indicates results for linear regression against GM volume scores alone, while the second and third line indicate results for models including both GM volume and LL as predictors.

| **NETWORK #** | **Parameter** | **B** | | | **SE** | **Standardized**  **B** | | | **p-value** |
| --- | --- | --- | --- | --- | --- | --- | --- | --- | --- |
| Network #1 | GM volume only | | 1.881 | 0.481 | | | 0.308 | **0.0001** | |
|  | GM volume | | 1.728 | 0.472 | | | 0.283 | **0.0003** | |
|  | LL | | -0.023 | 0.007 | | | -0.225 | **0.0011** | |
| Network #2 | GM volume only | | 8.348 | 2.082 | | | 0.337 | **0.0001** | |
|  | GM volume | | 6.882 | 2.119 | | | 0.278 | **0.0014** | |
|  | LL | | -0.020 | 0.007 | | | -0.192 | **0.0069** | |
| Network #4 | GM volume only | | 4.689 | 1.240 | | | 0.367 | **0.0002** | |
|  | GM volume | | 3.707 | 1.276 | | | 0.290 | **0.0041** | |
|  | LL | | -0.019 | 0.007 | | | -0.189 | **0.0085** | |
| Network #5 | GM volume only | | 72.180 | 18.277 | | | 0.346 | **0.0001** | |
|  | GM volume | | 58.143 | 18.783 | | | 0.279 | **0.0022** | |
|  | LL | | -0.019 | 0.007 | | | -0.187 | **0.0091** | |
| Network #6 | GM volume only | | 0.942 | 0.236 | | | 0.331 | **0.0001** | |
|  | GM volume | | 0.715 | 0.255 | | | 0.251 | **0.0056** | |
|  | LL | | -0.017 | 0.008 | | | -0.165 | **0.0287** | |
| Network #11 | GM volume only | | 5.232 | 1.291 | | | 0.318 | **0.0001** | |
|  | GM volume | | 4.789 | 1.268 | | | 0.291 | **0.0002** | |
|  | LL | | -0.023 | 0.007 | | | -0.222 | **0.0012** | |
| Network #12 | GM volume only | | 0.426 | 0.119 | | | 0.324 | **0.0004** | |
|  | GM volume | | 0.340 | 0.121 | | | 0.259 | **0.0053** | |
|  | LL | | -0.020 | 0.007 | | | -0.199 | **0.0053** | |
| Network #13 | GM volume only | | 5.850 | 1.475 | | | 0.316 | **0.0001** | |
|  | GM volume | | 5.472 | 1.442 | | | 0.295 | **0.0002** | |
|  | LL | | -0.024 | 0.007 | | | -0.230 | **0.0008** | |
| Network #14 | GM volume only | | 2.811 | 0.667 | | | 0.359 | **0.0000** | |
|  | GM volume | | 2.310 | 0.686 | | | 0.295 | **0.0009** | |
|  | LL | | -0.019 | 0.007 | | | -0.182 | **0.0108** | |
| Network #15 | GM volume only | | 0.456 | 0.123 | | | 0.267 | **0.0003** | |
|  | GM volume | | 0.293 | 0.157 | | | 0.172 | **0.0630** | |
|  | LL | | -0.015 | 0.009 | | | -0.147 | **0.0987** | |
| Network #16 | GM volume only | | 2.848 | 0.647 | | | 0.371 | **0.0000** | |
|  | GM volume | | 2.410 | 0.658 | | | 0.314 | **0.0003** | |
|  | LL | | -0.019 | 0.007 | | | -0.186 | **0.0083** | |
| Network #17 | GM volume only | | 73.177 | 20.056 | | | 0.336 | **0.0003** | |
|  | GM volume | | 57.387 | 20.588 | | | 0.264 | **0.0058** | |
|  | LL | | -0.020 | 0.007 | | | -0.193 | **0.0073** | |
| Network #18 | GM volume only | | 0.880 | 0.238 | | | 0.314 | **0.0003** | |
|  | GM volume | | 0.723 | 0.240 | | | 0.258 | **0.0029** | |
|  | LL | | -0.021 | 0.007 | | | -0.201 | **0.0045** | |
| Network #20 | GM volume only | | 10.172 | 2.682 | | | 0.295 | **0.0002** | |
|  | GM volume | | 7.972 | 2.776 | | | 0.231 | **0.0045** | |
|  | LL | | -0.019 | 0.007 | | | -0.187 | **0.0099** | |

LL: Lesion load (white matter).

**Table S4:** **Effect of disability level on models:** Statistical results from GM component models, exploring stratification by MS-related disability level, as measured by PDDS; outcome variable for all models was the Timed 25-foot walk normal walking speed. PDDS is only measured in MS participants. Disability level was dichotomized into low disability (PDDS = 0-2) vs high disability (PDDS= 3-5) groups. (Significant p-values are shown in bold, trend-level p-values are shown in yellow).

|  | **Low Disability Group (n = 55)** | | | | | | |  | **High Disability Group (n = 47)** | | | | | |  |
| --- | --- | --- | --- | --- | --- | --- | --- | --- | --- | --- | --- | --- | --- | --- | --- |
| **NETWORK #** | **B** | | **SE** | **Standardized B** | | | **p-value** | | **B** | **SE** | **Standardized B** | | | **p-value** | |
| Network #1 | 1.221 | 0.728 | | | 0.176 | **0.100** | | | 1.209 | 1.084 | | 0.186 | 0.271 | | |
| Network #2 | 5.862 | 2.710 | | | 0.224 | **0.035** | | | 2.592 | 4.096 | | 0.103 | 0.530 | | |
| Network #4 | 3.315 | 1.429 | | | 0.251 | **0.025** | | | -0.737 | 2.345 | | -0.054 | 0.755 | | |
| Network #5 | 39.582 | 21.100 | | | 0.202 | **0.067** | | | 24.997 | 34.345 | | 0.125 | 0.471 | | |
| Network #6 | 0.737 | 0.285 | | | 0.260 | **0.013** | | | -0.217 | 0.505 | | -0.071 | 0.670 | | |
| Network #11 | 3.420 | 1.837 | | | 0.192 | **0.069** | | | 3.083 | 2.837 | | 0.179 | 0.283 | | |
| Network #12 | 0.225 | 0.141 | | | 0.172 | 0.115 | | | 0.070 | 0.240 | | 0.050 | 0.773 | | |
| Network #13 | 3.352 | 2.019 | | | 0.172 | 0.103 | | | 3.345 | 3.331 | | 0.167 | 0.321 | | |
| Network #14 | 1.761 | 0.888 | | | 0.209 | **0.053** | | | 0.819 | 1.269 | | 0.107 | 0.523 | | |
| Network #15 | 0.324 | 0.179 | | | 0.184 | **0.076** | | | -0.227 | 0.256 | | -0.136 | 0.380 | | |
| Network #16 | 1.986 | 0.924 | | | 0.225 | **0.037** | | | 1.047 | 1.269 | | 0.136 | 0.414 | | |
| Network #17 | 62.020 | 24.625 | | | 0.270 | **0.015** | | | -17.126 | 39.718 | | -0.074 | 0.669 | | |
| Network #18 | 0.375 | 0.340 | | | 0.121 | 0.275 | | | 0.220 | 0.462 | | 0.080 | 0.636 | | |
| Network #20 | 4.906 | 3.659 | | | 0.143 | 0.186 | | | 10.576 | 6.094 | | 0.276 | **0.090** | | |
